# Effect of Lateralization, Age, and Sex on Frequency Following Response in Children: Neural Speech Encoding to a 170 ms [da] Stimulus

**DOI:** 10.3390/life16040695

**Published:** 2026-04-21

**Authors:** Caroline Donadon, Milaine Dominici Sanfins, Aline Buratti Sanches, Gabriele Libano de Souza Cardoso, Ayla Gabrielle Paschoalon de Mello, Piotr Henryk Skarzynski, Maria Francisca Colella-Santos

**Affiliations:** 1 Graduate Program in Child and Adolescent Health, Faculty of Medical Sciences, State University of Campinas, Campinas 13083-887, SP, Brazil; caroldonadon.fono@gmail.com (C.D.);; 2 Department of Speech-Hearing-Language, Universidade Federal de São Paulo, São Paulo 04044-020, SP, Brazil; 3 Department of Teleaudiology and Screening, World Hearing Center, Institute of Physiology and Pathology of Hearing, 05-830 Warsaw, Poland; 4 Post-Graduate Program in Clinical Audiology, Instituto de Ensino e Pesquisa Albert Einstein, São Paulo 05652-000, SP, Brazil; 5 Graduate Program in Health, Interdisciplinarity and Rehabilitation, Faculty of Medical Sciences, State University of Campinas, Campinas 13083-887, SP, Brazil; 6 Center of Hearing and Speech Medincus, 05-830 Warsaw, Poland; 7 Institute of Sensory Organs, 05-830 Warsaw, Poland; 8 Full Professor at Faculty of Medical Sciences, State University of Campinas, Campinas 13083-887, SP, Brazil

**Keywords:** frequency-following response, speech-evoked potentials, school-aged children, neural encoding

## Abstract

Central auditory processing efficiency is considered a predictor of how well children can learn to read, with the Frequency Following Response (FFR) serving as a sensitive biomarker of neural speech encoding ability. However, data regarding the 170 ms [da] stimulus in children who are native speakers of Brazilian Portuguese (BP) remain limited. This study investigated FFR results in 37 typically developing, normal-hearing children aged 8 to 10 years. Participants underwent audiological, behavioral, and academic performance screenings, followed by monaural FFR recording (using a 170 ms [da] stimulus at 80 dBnHL). Linear mixed models (LMM) were used to examine the effects of age, sex, and ear on the latencies of waves V, A, D, E, F, and O. The analysis revealed a medium effect size for waves D, E, and F, regarding the Ear factor, though statistical significance was specifically observed for wave E. For this wave, sex was also associated with a medium effect size, characterized by longer latencies in female participants. While the results for age did not reach broad statistical significance, the presence of medium effect sizes in wave E may suggest ongoing refinement of neural synchrony and asymmetric maturation during this developmental period. This study contributes to the characterization of neural speech encoding in the Brazilian Portuguese-speaking children and may support future investigation involving auditory processing disorders and learning difficulties.

## 1. Introduction

Auditory processing of verbal stimuli is a complex neurophysiological process involving the detection, analysis, and interpretation of acoustic signals. In school-aged children, the efficiency of this system is a direct predictor of success of literacy and speech understanding in noisy environments [1,2]. Auditory evoked potentials are the primary objective tools that allow neural transmission to be monitored, with an ability to diagnose hearing loss or impairments in the central auditory pathways [3]. Traditionally, these potentials are generated by non-verbal stimuli, such as clicks and chirps; however, such stimuli do not reflect the complex nature of the information transmitted by verbal sounds [3,4].

While a click stimulus evaluates neural synchrony in response to an abrupt onset, the Frequency Following Response (FFR), using short verbal stimuli provides a unique window into the processing of speech sounds. The FFR can be sensitive and objective biomarker of central auditory processing [5] due to its ability to reflect the acoustic characteristics of verbal sounds [6]. The literature describes the FFR as a “snapshot” of the fidelity with which the auditory system represents the spectro-temporal characteristics of sounds [7]. The FFR reflects the synchronous activity of multiple neural ensembles, and such activity is considered a major biological prerequisite for “perceptual narrowing”, the process by which the developing brain refines its sensitivity to native language phonetic contrasts [8]. Thus, the FFR serves as a predictive biomarker for the development of reading and writing skills [9,10].

Evidence indicates that the maturation of the subcortical auditory system continues throughout childhood [11]. Longitudinal studies indicate that FFRs become earlier, more robust, and more consistent with increasing age, reflecting the progressive myelination and synaptic refinement of the auditory pathway [12,13]. Understanding these developmental trajectories is important, particularly given that Brazilian Portuguese phonology exhibits language-specific rhythmic and spectro temporal features, which are known to shape neural speech encoding during development [14,15,16].

The most widely used stimulus in FFR protocols is the consonant-vowel (CV) syllable /da/ [7]. Different versions of this stimulus vary in duration, ranging from short forms (40 ms) to longer stimuli (170 ms) [6]. Longer stimuli bear a greater resemblance to natural speech, and their waveforms contain a richer array of temporal and spectral information (transients, harmonics, and prosodic variations), which are fundamental for following typical language development and the early identification of disorders [17,18,19].

Thus, the objective of this study was to investigate the FFR in typically developing, normal-hearing children who were native speakers of Brazilian Portuguese. We used a 170 ms consonant-vowel [da] stimulus and measured the absolute latencies of waves V, A, D, E, F, and O. Specifically, this study provides (i) a detailed characterization of absolute latencies for waves V, A, D, E, F, and O in this population; (ii) an analysis of the effects of age, sex, and ear using LMM, allowing for the examination of both fixed and random sources of variability; and (iii) a contribution to the understanding of neural speech encoding in a language-specific context, forming a benchmark that may support future clinical and research applications in auditory processing and learning-related disorders.

## 2. Materials and Methods

The experimental design followed a cross-sectional observational analytical model, approved by the Research Ethics Committee of University of Campinas (approval No. 3,349,549). The study was conducted at the Audiology Laboratories of the Department of Human Development and Rehabilitation between August 2021 and January 2023.

### 2.1. Participants and Selection Process

Volunteers were recruited from a state public school located on the university campus. The selection process was rigorous to ensure sample homogeneity regarding typical development. A total of 37 subjects were evaluated, aged between 8 and 10 years.

To be included, participants were required to present:

- Pure tone hearing thresholds ≤15 dB HL across all frequencies from 250 to 8000 Hz [20].
- Normal tympanic-ossicular system, evidenced by Type A tympanometric curves and the presence of ipsilateral acoustic reflexes [21].
- Absolute latency values for waves I, III, and V, as well as interpeak intervals I–III, III–V, and I–V, within the normal range for the age group in Auditory Brainstem Response (ABR) assessment using click stimuli [22].
- Academic performance classified as “average” or “above average” in the School Performance Test (Teste de Desempenho Escolar—TDE), confirming the absence of significant pedagogical difficulties [23].

Strict exclusion criteria were applied to individuals with a history of neurological disorders, language impairments, learning complaints, or recurrent otological history, as determined by a structured interview with parents or guardians.

### 2.2. Behavioral Auditory Processing Screening

The behavioral battery was essential to confirm that the neural encoding observed in the FFR corresponded to a functionally healthy central auditory system. The tests were conducted in an acoustic booth using an AC40 audiometer (Interacoustics, Middelfart, Denmark).

#### 2.2.1. Dichotic Digits Test (DDT)

The binaural integration test utilized lists of disyllabic digits in Brazilian Portuguese. Participants repeated four digits presented simultaneously (two in each ear). Normality was defined as 85% for the right ear and ≥82% for the left ear at age 8, and ≥95% for both ears from age 9 onwards [24].

#### 2.2.2. Random Gap Detection Test (RGDT)

Temporal resolution was measured by detecting silent intervals (gaps) between pairs of pure tones. The threshold was the average of the shortest gaps detected at 500, 1000, 2000, and 4000 Hz. The adopted normality criterion was ≥10 ms [25].

#### 2.2.3. Synthetic Sentence Identification (SSI) Test

Auditory closure ability was evaluated with an ipsilateral competitive message (signal-to-noise ratio: −15 dB). The subject identified written sentences while listening to a competing narrative. The minimum expected performance was 60% correct [24].

### 2.3. Educational Achievement Assessment

To ensure typical development and the absence of learning disabilities, all participants were evaluated using the School Performance Test (TDE). This standardized instrument assesses reading and writing skills. Only children with “average” or “above average” scores for their age and grade level were included in the study [23].

### 2.4. Electrophysiological Assessment

Assessments were performed using the SmartEP software version 2.70 (Intelligent Hearing Systems—IHS, Miami, FL, USA) in an acoustic and electrically shielded environment. Subjects remained seated in a comfortable reclining chair and were instructed to keep their eyes closed during the evaluation to avoid artifacts.

#### 2.4.1. Click-Evoked ABR

The ABR was used to ensure the integrity of the auditory pathway. The acoustic stimulus consisted of a click (rarefaction polarity, 80 dB nHL, 19.3/s, 0.1 ms duration) presented via ER-3A insert earphones. A total of 2000 stimuli were collected in two separate series to ensure reproducibility. Skin preparation was involved the use of abrasive paste, and surface electrodes were attached using electrolytic paste and micropore tape. The electrodes were placed following a two-channel montage: forehead (Fz), right (M2) and left (M1) mastoids, and ground (Fpz), with a Y-adaptor to the positive inputs connecting the Fz between channels A and B. Impedances were maintained below 3 kΩ.

#### 2.4.2. FFR

The stimulus consisted of a synthesized 170 ms [da] syllable provided by the SmartEP software (Intelligent Hearing Systems, Miami, FL, USA). This stimulus comprises a 50 ms transient period and a 120 ms sustained portion. The acoustic architecture of the stimulus included an initial burst and a 50 ms formant transition, in which the lower formants vary (F1: 725 Hz; F2: 1230 Hz; F3: 2500 Hz), followed by 120 ms of a steady-state vowel (/a/) with an F0 of 100 Hz.

Electrodes were placed following a two-channel montage: active electrode was positioned at Fz and connected via a Y-adaptor to the positive inputs of both channels (A and B). Reference electrodes were placed on the right (M2) and left (M1) mastoids, and the ground electrode was positioned at Fpz.

The stimulus was presented monaurally via ER-3A insert earphones at 80 dB nHL, using alternating polarity to minimize artifacts and enhance the neural response to the envelope. The presentation rate was 4.35/s, with an analysis window of 230 ms (including a 20 ms pre-stimulus period). Signal amplification was set at 100 K gain, with an online band-pass filter of 50–3000 Hz and an artifact rejection level of ±35 µV. Each recording session lasted approximately 30 min, ensuring at least 4000 valid sweeps per subject.

Data processing and analysis were realized with Complex ABR research module for SmartEP software (Intelligent Hearing Systems, Miami, FL, USA). For each subject, two sub-averages of 2000 valid sweeps were combined to generate a grand average waveform based on 4000 stimuli. This waveform was subsequently processed using an offline digital filter (70–2000 Hz), and the FFR peaks (V, A, D, E, F and O) were then manually identified by experienced examiners.

**Figure 1:**
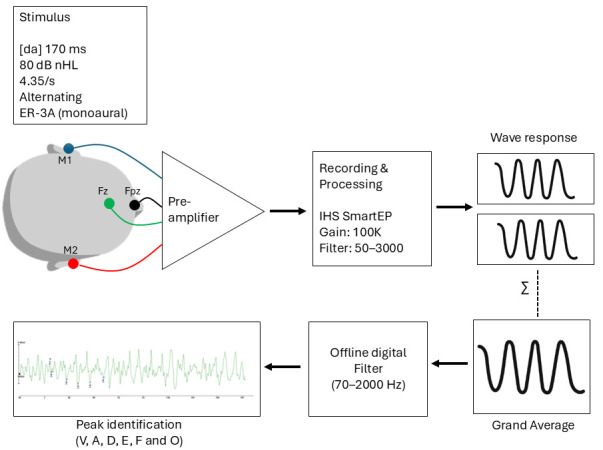
Schematic representation of the experimental setup for FFR recording. Ms, Milliseconds; dB nHL, decibel normalized hearing level; Fz, forehead; Fpz, ground; M1, left mastoid; M2, right mastoid; Hz, hertz; ∑, summation.

Overall, the data processing pipeline followed a structured sequence including signal acquisition, artifact rejection, averaging, offline filtering, and waveform peak identification (Figure 1), ensuring consistency and reproducibility of the electrophysiological measurements.

### 2.5. Statistical Analysis

Statistical analyses were performed using LMM to account for the hierarchical structure of the data and the inherent correlation between observations within the same subject. This approach was selected because it allows for the inclusion of correlated measures and random effects, which are relevant for modeling inter-subject variability [26]. Furthermore, LMM are considered resistant to violations of the assumptions commonly associated with traditional parametric analyses [27].

In the model, age, sex, and ear (left vs. right), as well as the interaction between ear and age, were included as fixed effects. Subjects were included as random effects (intercepts) to account for individual variability. This modeling approach also allows the independent contribution of each predictor variable to be estimated while controlling for the effects of the others. Each ear was treated as a repeated measure within subjects, and this dependency was accounted for in the LMM structure.

In addition to *p*-values, the results were interpreted based on effect size, as they provide a more informative estimative of the magnitude of the observed effects, regardless of sample size [28,29]. Analysis was conducted using jamovi (version 2.4.11) with the GAMLj module [30] for R (version 4.1). Descriptive statistics and general data management were performed using SPSS Statistics (version 27.0). This approach supports computational transparency and appropriate handling of correlated FFR data.

Effect sizes were interpreted according to the criteria proposed by Cohen [31]. Post hoc analyses with Bonferroni correction were conducted to explore significant interactions [27,31]. The significance level was set at 5%.

## 3. Results

The sample consisted of 37 typically developing children (mean age 9.13 ± 0.85 years). As shown in Table 1, all participants exhibited age-appropriate academic performance (TDE) and met normality criteria for all behavioral auditory processing measures (DDT, RGDT, and SSI).

### 3.1. FFR Wave Latencies

Absolute latencies for both transient and sustained waves were recorded for all children. Table 2 details these values, providing the first comprehensive normative dataset for the 170 ms stimulus in this population.

### 3.2. Effects of Sex, Ears, and Age

The LMM analysis revealed complex interactions between factors (Table 3). A statistically significant effect of Ear was observed for Wave E latency (*p* = 0.025), along with larger effect sizes for Ear × Age interaction across waves V, A, D, E, and F.

Although the *p*-value significance did not reach statistical significance except for wave E, where *p* = 0.025 for Ear as a factor. However, the effect size (E.S.) suggested there may be medium practical impacts for the Ear factor in waves D, E, and F, and for the Age factor in wave E.

Beyond the identification of these specific effects, we sought to understand the potential hierarchy of importance among the predictors. Analysis of the *F*-statistics appears to reveal a trend where Ear emerges as a more consistent driver of neural encoding. Based on the relatively higher *F*-values for Ear in waves D (*F* = 3.784), E (*F* = 5.521), and F (*F* = 2.334) compared to Gender and Age, it could be hypothesized that functional asymmetry is a prominent feature in shaping neural speech encoding in this population, potentially outweighing the immediate impacts of chronological age or gender in this specific cohort.

Post hoc analyses (Table 4) revealed that at age 8, the right ear exhibited generally longer latencies for waves V, A, E, and F, while the left ear was slower for wave D, although none reached statistical significance. At age 9, a tendency towards longer latencies in the right ear persisted for waves A, E, and F.

## 4. Discussion

The present investigation suggests that neural speech encoding in school-aged children is a dynamic process, influenced by the asymmetrical maturation of auditory pathways and the spectro-temporal characteristics of the acoustic stimulus. This analysis provides insights into the maturation of auditory pathways, functional lateralization, and the potential influence of the Brazilian Portuguese language on neural synchrony.

### 4.1. Effects of Maturation and the Age Factor

The maturation of the subcortical auditory pathway is a protracted process that extends well into the first decade of life and beyond [32]. This developmental trajectory is characterized by the progressive myelination of auditory fibers and the refinement of synaptic transmission, which collectively increase nerve conduction velocity and temporal precision [11].

Age appears to have an impact on wave latencies, particularly on wave E, suggesting that the maturation of subcortical auditory pathways between 8 and 10 years of age may influence on the encoding of the sustained portion of speech, reflecting a progressive refinement of neural synchrony [33]. Longitudinal studies, such as those by Thompson et al. [1], indicate that subcortical auditory maturation occurs progressively, contributing to increased stability and consistency of the FFR over time. Ferreira et al. [11] observed that older children tend to present more stable and organized FFRs than younger ones, with a significant reduction in latencies as development progresses.

The present findings are consistent with these observations, suggesting that fine-tuning in the encoding of the temporal structure of speech (/da/stimulus) may continue until age 10. This refinement may be associated with the consolidation of complex skills, such as reading and speech comprehension in noisy environments.

The reduction in latencies observed in childhood appears to be mirrored by the decline seen in aging populations. Relationships between age and FFR results have also been documented in adults, although in the opposite direction of decline. Clinard et al. [34], evaluating adults aged 22–77 years with the 40 ms [da] syllable, reported that FFR components are negatively affected by aging, showing reduced response synchrony and amplitude, as well latency prolongation associated with aging. Similarly, Anderson and Kraus [35], using the 170 ms [da] stimulus, observed a decrease in the robustness of frequency representation and in the amplitude of the fundamental frequency (F0) with advancing age.

The relative stabilization of absolute latencies in the sustained portion (waves D, E, F, and O) in children aged 8–10 years may indicate that the encoding of the temporal envelope and fine structure of speech sounds is becoming well established by this stage. This pattern may reflect increased nerve conduction velocity [36], likely associated with the progressive myelination of auditory fibers [37]. Such maturation may support a more faithful neural representation of the acoustic stimulus, which is relevant for complex academic tasks such as reading and classroom listening.

### 4.2. Laterality and the Ear × Age Interaction

Previous studies indicate that neural refinement during childhood does not follow a strict linear pattern, showing variability related to the hemisphere specialization and individual differences in auditory maturation [38]. This variability may help explain the differences observed in latencies patterns across specific waves and ages in the present sample.

One of the main findings of this study was the influence of the Ear × Age interaction on waves V, A, D, E, and F, suggesting that the asymmetry between ears may evolve with development. The longer latencies observed in the right ear for waves V, A, E, and F at ages 8 and 9, followed by a trend toward reduction and stabilization at age 10, may reflect aspects of the developmental trajectory of hemispheric dominance for language.

Although the left hemisphere predominantly receives input from the right ear and is specialized in processing rapid temporal aspects of speech [11], the subcortical pathways supporting this connection may still be undergoing refinement during early school aged [37]. The detection of longer latencies in the right ear may suggest that the system is still undergoing fine-tuning to process the acoustic complexity of the 170 ms stimulus, possibly requiring further maturational adjustments in the pathways projecting to the dominant hemisphere.

The finding that wave D (formant transition) presented longer latencies in the left ear at ages 8 and 10 supports the notion that the central auditory system processes different speech components in a lateralized manner. These findings may suggest that the right pathway could be relatively more efficient in processing complex linguistic stimulus, while the left pathway may be more involved in encoding prosodic and non-linguistic aspects [39]. This subcortical specialization may provide a biological basis for the right-ear advantage frequently observed in behavioral dichotic tests, reflecting the hierarchical and functional organization of the central auditory system [40,41].

Beyond these interaction effects, the analysis of predictors within the LMM suggests that Ear factor may represent a more consistent contributor to neural encoding variability in this cohort than other demographic variables. Based on the relatively higher *F*-statistics observed for the ear factor across waves D, E, and F, functional asymmetry may play an important role in neural speech encoding stability in children. This interpretation is consistent with cross-linguistic evidence indicating that subcortical lateralization may be influenced by early linguistic exposure and auditory specialization [8]. When considered independently within the LMM framework, the effect of ear remained the most consistent predictor of FFR latency variability, whereas age effects were more restricted to specific components and sex effects were minimal.

### 4.3. Influence of Sex on Wave E Encoding

Although the previous studies have reported functional and structural differences in the auditory system between males and females [42], indicating that auditory evoked potentials typically exhibit longer latencies and smaller amplitudes in males compared to females [43], the present results showed slightly longer latencies for wave E in female participants. It is possible that the encoding of vowel periodicity (wave E) may follow distinct maturational trajectories between sexes, potentially influenced by varying estrogen levels or the rate of synaptic development in the inferior colliculus [43].

The divergence between these findings and the existing literature may suggest that the encoding characteristics of the [da] syllable in childhood may not necessarily follow the patterns described in adult populations. Additionally, the use of a long stimulus (170 ms) may have allowed for the observation of subtle aspects of fundamental frequency (F0) processing that shorter stimulus (e.g., 40 ms) may not capture with the same sensitivity.

### 4.4. Latency Stability

The stability of absolute latencies observed in the sustained portion (D, E, F, and O) suggests that the encoding of the temporal envelope and fine structure of speech sounds may be relatively well established in children aged 8 to 10 years. This finding is consistent with the low data variability, indicating that the subcortical auditory system may have reached a level of maturation that supports synchronous and stable neural responses to complex acoustic stimuli. Such robustness may reflect a functionally efficient auditory system capable of sustaining the phase-locking mechanisms required for speech processing [13,44,45].

### 4.5. Comparative Analysis of Latencies for the 170 ms [da] Stimulus

The use of the 170 ms stimulus appears to be an effective in eliciting all FFR components with good reproducibility in Brazilian children. The relatively low variability observed, reflected in consistent standard deviations, suggests sample homogeneity and supports the stability of neural encoding in this population. Neural speech encoding is not solely a biological phenomenon; it may also be shaped by the linguistic environment of the listener [46].

Brazilian Portuguese (BP) exhibits phonological and acoustic characteristics that may differ from those described for English, which has been extensively studied in FFR research [6]. BP is often described as presenting a relatively “melodic” prosodic profile, with a predominance of open vowels and specific rhythmic patterns [47], which may be associated with differences in neural phase-locking mechanisms [15,16]. These phonetic acoustic characteristics could potentially facilitate phase-locking to the fundamental frequency (F0) and its harmonics [48]. Taken together, these considerations suggest the relevance of cross-linguistic comparisons, as variability in FFR latencies may reflect not only developmental and methodological factors but also, at least in part, language-specific influences on neural speech encoding [49,50].

The present findings were interpreted in relation to previously reported FFR latency patterns in both pediatric and adult populations, allowing the results to be contextualized within a broader developmental and cross-linguistic framework. Previous studies have shown that FFR latencies tend to decrease with maturation and remain relatively stable in young adults, reflecting progressive refinement of neural synchrony [13,51]. In addition, language-dependent modulation of FFRs has been described [4], supporting the notion that neural speech encoding reflects both biological maturation and auditory experience.

The mean values obtained for the sustained components (D, E, F, and O) show general agreement with those reported in normal-hearing populations [36,52]. However, differences were observed in the onset responses (V-A complex), with longer latencies for wave V and shorter latencies for wave A compared to previous studies. Comparisons with White-Schwoch et al. [53] revealed reduced latencies for waves A, F, and O, while other components (V, D, and E) remained similar. In contrast, Hornickel et al. [54] reported comparable values for most waves, except for wave V, which showed longer latencies. Similar patterns have also been described in young adults [52,55], suggesting that the key aspects of neural speech encoding may reach relative stability early in development.

Taken together, these comparisons allow the present findings to be positioned relative to previously reported datasets, highlighting both convergences and differences that may be associated with developmental stage, stimulus parameters, and linguistic background.

### 4.6. Clinical Utility and the Future of Automated Diagnostics

The FFR has been proposed as a potential biomarker for conditions, such as auditory processing disorders (APD), learning disabilities, and dyslexia [45,56]. Children with these conditions often exhibit prolonged latencies or reduced response consistency, which may reflect underlying deficits in neural synchrony [4,57]. In addition to reinforcing the stability of FFR components in typically developing children, these results support the use of speech-evoked auditory potentials as sensitive tools for investigating subtle variations in neural synchrony. The relatively low variability observed in the sustained components further highlights the potential of these measures as reliable markers of auditory function

From a clinical perspective, these data could potentially be utilized as an objective biomarker in the diagnosis and monitoring of CAPD. For instance, since the 170 ms stimulus captures a more sustained portion of the vocalic transition, it may be particularly useful in evaluating children who struggle with speech perception in noise. Furthermore, these data could serve as a baseline for measuring the efficacy of acoustically controlled auditory training, allowing clinicians to track objective changes in subcortical speech encoding over time.

Furthermore, the integration of FFR-derived parameters into machine learning (ML) frameworks may represent a potential direction for future research in audiology. Classification approaches, including the use of confusion matrices, may support the development of automated tools capable of distinguishing typical from atypical auditory profiles [58]. The high temporal resolution of the FFR, combined with the relative stability of sustained components, may suggest its potential suitability for such data-driven applications.

In addition, recent advances in computational approaches have explored the robustness of auditory signal processing under conditions of perturbation and noise. Studies investigating adversarial manipulations in speech recognition systems have demonstrated that subtle alterations in acoustic signals can significantly affect classification performance, highlighting the sensitivity of auditory models to fine-grained temporal and spectral variations [59,60,61].

Although these approaches are primarily developed within machine learning frameworks, they may offer complementary perspectives for understanding the robustness of neural speech encoding. In this context, electrophysiological measures such as the FFR may provide relevant insights into how the human auditory system responds to variations or distortions in acoustic input.

## 5. Conclusions

This study characterized the FFR using a 170 ms [da] stimulus in Brazilian Portuguese-speaking children aged 8 to 10 years. The findings suggest that subcortical auditory maturation during this period may be influenced by factors such as ear and, to a lesser extent, age. Differences in latency between ears may reflect aspects of asymmetrical maturation of auditory pathways and the ongoing development of hemispheric specialization. These results may contribute to the understanding of auditory function in this population and may support future investigations, including studies involving auditory processing disorders and learning difficulties.

Some limitations should be considered when interpreting the results. The sample size, although homogeneous, was relatively small, and the cross-sectional design and the specific age range (8 to 10 years) suggest that these results should be interpreted with caution and may not be fully representative of other developmental stages.

Future studies may benefit from longitudinal designs to better characterize maturational changes over time, as well as from the inclusion of clinical populations to explore the diagnostic applicability of FFR measures. Additionally, exploring the impact of different sociolinguistic backgrounds and comparing these normative data with clinical populations (e.g., children with dyslexia or ADHD) would be essential to further validate the clinical utility of the 170 ms [da] stimulus. Further investigations examining different speech stimuli and cross-linguistic comparisons may also contribute to a deeper understanding of the interaction between auditory development and linguistic experience.

## Figures and Tables

**Table 1:** Demographic, educational, and behavioral auditory characteristics of the study sample.

Variable		Total (N = 37)	8 Years (n = 12)	9 Years (n = 12)	10 Years (n = 13)
**Demographics**					
Sex (Male/Female)		21/16	8/3	6/4	5/8
Age (Mean ± SD)		9.13 ± 0.85	8.16 ± 0.17	9.13 ± 0.32	10.03 ± 0.05
**Educational Performance (TDE)**					
Reading Score (Mean ± SD)		66.59 ± 2.73	63.73 ± 2.61	67.50 ± 1.35	68.38 ± 1.41
Writing Score (Mean ± SD)		28.36 ± 4.47	24.00 ± 4.36	28.70 ± 2.58	32.06 ± 1.61
**Auditory Processing Screening**					
** DDT (%)**	**(Mean ± SD)**				
Right Ear		97.23 ± 3.03	95.68 ± 3.72	98.50 ± 2.11	97.50 ± 2.58
Left Ear		96.34 ± 4.95	94.09 ± 6.55	98.00 ± 2.30	96.88 ± 4.61
** SSI (ICM −15) (%)**	**(Mean ± SD)**				
Right Ear		74.88 ± 11.76	70.91 ± 8.31	76.00 ± 14.30	77.50 ± 11.83
Left Ear		74.58 ± 12.07	70.00 ± 10.95	76.00 ± 15.06	77.50 ± 9.31
** RGDT (ms)**	**(Mean ± SD)**				
Both ears		6.35 ± 2.12	5.86 ± 1.99	6.60 ± 1.81	6.56 ± 2.55

**Legend**: N, total number of participants; n, number of participants per group; SD, Standard Deviation; TDE, School Performance Test (Teste de Desempenho Escolar); DDT, Dichotic Digits Test; RGDT, Random Gap Detection Test; SSI, Synthetic Sentence Identification; ICM, Ipsilateral Competitive Message. **Note:** All participants met the normality criteria for their respective age groups.

**Table 2:** FFR wave latencies by sex, age, and ear (values in ms).

Wave	Sex	Ear	8 Years (n = 12)				9 Years (n = 12)				10 Years (n = 13)			
			M	SD	Min	Max	M	SD	Min	Max	M	SD	Min	Max
	Female	RE	8.35	0.48	7.80	8.63	8.43	0.23	8.10	8.63	7.63	0.25	7.05	7.88
V		LE	8.00	0.78	7.35	8.86	8.52	0.76	7.88	9.60	7.53	0.33	6.83	7.88
	Male	RE	8.05	0.98	6.97	9.75	7.86	0.38	7.50	8.47	8.02	1.05	7.28	10.28
		LE	7.96	1.04	6.60	10.13	7.97	1.03	6.97	9.97	8.37	1.02	7.50	10.28
	Female	RE	10.47	0.31	10.13	10.72	10.87	0.92	9.60	11.63	9.65	0.79	8.63	10.88
A		LE	9.90	0.81	9.00	10.57	10.03	0.52	9.47	10.72	9.86	1.09	8.47	11.25
	Male	RE	10.26	0.78	9.07	11.47	10.28	0.50	9.60	10.88	10.20	1.00	9.00	11.63
		LE	10.06	0.90	8.65	11.47	10.08	1.27	8.85	12.22	10.18	1.15	8.85	12.15
	Female	RE	24.30	0.86	23.63	25.27	24.39	0.78	23.40	25.12	24.12	0.54	23.40	24.90
D		LE	24.84	1.05	23.63	25.50	25.10	0.70	24.20	25.65	24.20	0.58	23.25	24.75
	Male	RE	24.49	0.66	23.70	25.27	24.38	0.74	23.33	25.50	24.24	1.62	21.07	26.02
		LE	25.17	1.29	23.40	27.15	24.20	0.67	23.02	24.90	24.73	0.72	23.55	25.95
	Female	RE	34.60	1.65	33.00	36.30	35.56	1.48	34.50	37.70	34.44	1.38	32.63	37.35
E		LE	34.23	1.72	32.40	35.80	34.68	0.59	34.27	35.55	34.27	0.92	33.15	36.15
	Male	RE	34.82	0.79	33.38	36.08	35.27	0.78	34.27	36.15	34.14	1.17	32.48	35.63
		LE	34.46	0.86	33.00	35.63	34.13	0.72	33.00	35.25	33.83	0.70	33.00	34.68
	Female	RE	44.10	1.24	42.67	44.85	45.02	0.77	44.17	45.67	44.26	0.91	42.90	45.67
F		LE	44.36	0.81	43.42	44.85	44.24	0.47	43.65	44.77	44.23	0.76	42.67	45.15
	Male	RE	44.79	0.41	44.17	45.30	44.37	0.65	43.42	45.15	44.12	1.03	42.23	45.30
		LE	44.14	0.54	43.05	44.92	43.98	1.24	42.30	45.90	44.36	0.99	42.90	45.90
	Female	RE	55.35	2.73	52.42	57.83	55.47	1.59	54.08	57.65	54.67	1.74	52.58	57.08
O		LE	55.64	2.58	52.80	57.83	56.14	1.02	54.92	57.30	54.58	1.91	52.58	58.95
	Male	RE	55.16	2.51	53.40	61.20	54.95	1.24	53.48	56.92	55.04	3.10	52.73	61.05
		LE	54.93	2.23	52.20	58.58	54.61	1.71	52.95	56.92	54.71	2.54	52.95	59.25

**Legend**: n, number of participants per group; SD, Standard Deviation; M, Mean; Min, Minimum; Max, Maximum; RE, Right Ear; LE, Left Ear.

**Table 3:** Tests of Fixed Effects for FFR Wave Latencies.

Factor		V	A	D	E	F	O
(df1, df2)							
	F	0.104	0.210	0.131	0.825	0.114	0.194
Gender	*p*	0.749	0.650	0.720	0.370	0.738	0.662
(1.33)	E.S.	0.056	0.078	0.089	0.215 ^†^	0.059	0.075
	F	0.014	1.633	3.784	5.521	2.334	0.056
Ear	*p*	0.905	0.210	0.060	0.025 *	0.136	0.814
(1.34)	E.S.	0.029	0.296 ^†^	0.321 ^††^	0.374 ^††^	0.352 ^††^	0.057
	F	0.471	0.581	1.126	2.948	0.276	0.300
Age	*p*	0.629	0.565	0.337	0.066	0.760	0.743
(2.33)	E.S.	0.119 ^†^	0.130 ^†^	0.253 ^†^	0.384 ^††^	0.091	0.094
	F	0.479	1.134	0.563	1.123	1.161	0.071
Ear × Age	*p*	0.624	0.333	0.575	0.337	0.325	0.932
(2.34)	E.S.	0.168 ^†^	0.250 ^†^	0.130 ^†^	0.179 ^†^	0.256 ^†^	0.064

**Legend**: *, statistically significant value at 5% level; df, Degrees of Freedom; E.S., Effect Size; ^†^, small effect; ^††^, medium effect.

**Table 4:** Pairwise comparisons for ear effects on FFR wave latencies across age groups.

Wave	Age	Difference	SE	*t*-Value	*p*-Value	E.S.
	8	−0.16	0.22	−0.727	>0.999	0.124 ^†^
V	9	0.10	0.23	0.435	>0.999	0.074
	10	0.10	0.18	0.556	>0.999	0.095
	8	−0.30	0.30	1.000	0.973	0.169 ^†^
A	9	−0.46	0.32	1.438	0.479	0.239 ^†^
	10	0.11	0.25	0.440	>0.999	0.075
	8	0.64	0.33	1.939	0.182	0.316 ^††^
D	9	0.18	0.35	0.514	>0.999	0.088
	10	0.26	0.28	0.929	>0.999	0.157 ^†^
	8	−0.36	0.42	0.857	>0.999	0.145 ^†^
E	9	−1.04	0.44	2.364	0.072	0.376 ^††^
	10	−0.23	0.34	0.676	>0.999	0.115 ^†^
	8	−0.40	0.34	1.176	0.743	0.198 ^†^
F	9	−0.54	0.35	1.543	0.396	0.256 ^†^
	10	0.09	0.28	0.321	>0.999	0.055

**Legend**: SE, Standard Error; E.S., Effect Size; ^†^, small effect; ^††^, medium effect. Notes: df = 34 was used for all comparisons. Student’s *t*-test with Bonferroni correction was applied.

## Data Availability

The original contributions presented in this study are included in the article. Further inquiries can be directed to the corresponding author.

## Abbreviations

The following abbreviations are used in this manuscript:

FFR	Frequency following response
ABR	Auditory brainstem response
ADHD	Attention Deficit Hyperactivity Disorder
APD	auditory processing disorders
TDE	School Performance Test (Teste de Desempenho Escolar)
SD	Standard Deviation
LMM	Linear Mixed Models
BP	Brazilian Portuguese
ms	Milliseconds
ML	Machine Learning
Hz	Hertz
dB HL	Decibels Hearing Level
IHS	Intelligent Hearing Systems
CNPq	National Council for Scientific and Technological Development
DDT	Dichotic Digits Test
RGDT	Random Gap Detection Test
SSI	Synthetic Sentence Identification
CV	Consonant-Vowel
F0	Fundamental frequency
F1	Frist formant
F2	Second formant
F3	Third formant
nHL	Normalized Hearing Level
KΩ	kiloohm
